# Investigation of the blood proteome in response to spinal cord injury in rodent models

**DOI:** 10.1038/s41393-021-00692-8

**Published:** 2021-10-02

**Authors:** Charlotte H. Hulme, Heidi R. Fuller, John Riddell, Sally L. Shirran, Catherine H. Botting, Aheed Osman, Karina T. Wright

**Affiliations:** 1 School of Pharmacy and Bioengineering, Keele University, Keele, Staffordshire, UK; 2 Midlands Centre for Spinal Injuries, RJAH Orthopaedic Hospital, Oswestry, Shropshire UK; 3 Institute of Neuroscience and Psychology, University of Glasgow, Glasgow, UK; 4 BSRC Mass Spectrometry and Proteomics Facility, University of St Andrews, North Haugh, St Andrews, UK

**Keywords:** Preclinical research, Biological techniques

## Abstract

**Study design:**

Explanatory and mechanistic study.

**Objectives:**

A better understanding of the ‘whole-body’ response following spinal cord injury (SCI) is needed to guide future research aimed at developing novel therapeutic interventions and identifying prognostic indicators for SCI. This study aimed to characterise the blood proteome following contusion or complete SCI compared to a sham injury in rat models.

**Setting:**

United Kingdom.

**Methods:**

Pooled blood samples from one and seven days after a contusion (serum; *n* = 5) or from 14 days and 112 days post-complete transection SCI (plasma; *n* = 8) and their sham-injured counterparts were subjected to independent iTRAQ nanoflow liquid chromatography tandem mass-spectrometry proteomic analyses. Pathway analyses of the proteins that were differentially abundant between SCI and their matched sham injured counterparts were completed to indicate biological pathways that may be changed in response to SCI.

**Results:**

Eleven and 42 proteins were differentially abundant (≥±2.0 FC; *p* ≤ 0.05) between the contusion SCI and sham injured animals at 24 h and seven days post-injury, respectively. Seven and tweleve proteins were differentially abundant between complete and sham injured rats at 14 and 112 days post-injury, respectively. Acute-phase response signalling and Liver X Receptor/Retinoic X Receptor activation were identified as differentially regulated pathways in both models of SCI.

**Conclusions:**

We have utilised longitudinal preclinical SCI models to provide an insight into the blood proteome changes that result following SCI and to highlight a number of biological pathways of interest for future studies.

## Introduction

Many clinical and scientific attempts have been made to better diagnose and to develop new treatments to improve the prognosis of individuals following a spinal cord injury (SCI). There is growing evidence that measures of some haematological indices post-injury relate with longer term (12 month) clinical outcome measures in human SCI patients [1, 2]. These studies indicate that over-time the ‘whole-body’, multi-organ response to SCI contributes to long-term clinical outcome and that proteins within the blood can be used to assess this phenomenon [1, 2]. Therefore a better understanding of the longitudinal protein changes within the blood following a SCI is needed to develop novel interventions [3].

The majority of studies that have aimed to understand protein changes that occur following a SCI, have focused upon changes within the SC tissue itself [3]. We have previously reviewed the few studies that have assessed the protein response to SCI in either the blood or cerebrospinal fluid (CSF) of SCI animal models or humans with a SCI [3]. It is particularly desirable to identify markers that can be measured systemically as blood samples can be collected immediately after injury with fewer associated risks compared to CSF samples. Having a better understanding of the proteome response to SCI in blood, will aid in the development of novel inteventions for SCI, as well as to identify candidate biomarkers that may have the potential to better determine SCI severity or predict clinical outcome of human with a SCI [4].

Several well-characterised pre-clinical SCI models exist which aim to represent the different common human SCI situations, contusive (blunt force) or penetrative (stab) injuries. In animals contusion injuries are commonly modelled using specialised devices that deliver an impact of calibrated force to the surgically exposed spinal cord [5, 6]. Penetrative injury, in humans more commonly leads to complete SCI, in which a variable cross section of the spinal cord is transected [7]. Surgical exposure and complete incision across all SC tracts is commonly performed in these animal models using either microscissors or a scalpel blade under visual control [8]. Rodent models are invaluable in studying the underlying mechanisms of SCI, as there is evidence that they undergo similar biological processes to humans following injury [9] and allow for comparison to sham injured animals (via surgical exposure of, but no injury to, the SC) whereas such comparator control groups are difficult to identify in the human situation.

Proteomics is a powerful analytical tool that can be used to profile large numbers of proteins in an untargeted manner. We have assessed the blood proteome of rat models of both a contusion and a complete transection SCI to determine whether we can detect differences between SC injured and sham injured rodents. Consideration of the blood proteome in such pre-clinical injury models could provide a more in-depth understanding of the biochemical response to SCI compared to sham injury and how these responses change over time in each independent model.

## Methods

### Animals used in the study

All experimental procedures were approved by the Ethical Review Panel of the University of Glasgow and carried out in accordance with the Animals (Scientific Procedures) Act 1986 and adhering to our recommended good practice for SCI rodent models [10]. Blood samples were collected surplus to the study outcomes and were used in this study to maximise the output from animal experiments in the spirit of the 3R’s of animal use (replacement, refinement and reduction) [11]. Blood samples were obtained at 1 day (*n* = 5) or 7 days (*n* = 5) after a contusion injury performed at the C6 level [12] or at 14 days (*n* = 8) or 112 days (*n* = 8) after a complete spinal cord transection performed at the T9 level [13]. Control blood samples were obtained from animals that underwent sham surgery performed at the same spinal level. Further details of the animal experiments are included in the supplementary material (appendix 1).

### Isobaric tag for relative and absolute quantitation proteomics

Two independent proteomic experiments were performed; one for the contusion SCI (serum samples) and the other for the complete SCI (plasma samples) models. Briefly, samples were pooled from the different injury and timepoint groups, then prepared and analysed using isobaric tagging for relative and absolute quantitation (iTRAQ) proteomics, as described previously [14] and detailed in the supplementary material (Appendix 1). Proteins which were differentially abundant between the SCI and matched sham counterparts were selected where *p* < 0.05 and the fold change (FC) was ≥±2.0.

### Pathway analysis of proteomic datasets

Proteins were analysed using the pathway enrichment and topological analysis tools in Ingenuity (Qiagen, US) [15] to identify and visualise the canonical pathways which are differentially effected between SCI and sham injured rats at each of the timepoints of the study and in the different SCI models. Functional annotations that were assigned a *p* value > 0.05, as assessed using a Fisher’s exact test were removed from the list.

## Results

### Differential abundance of proteins in the contusion versus sham injured animals

All the proteins identified in the proteomic analysis of serum from contusion SCI or sham counterparts are included in supplementary proteomic Table 1. Eleven proteins demonstrated ≥2.0 fold differential abundance in the serum of contusion SC injured compared to sham injured rats at one day post-injury (Table 1). At seven days post-injury 42 proteins were differentially abundant between contusion and sham injured rodents (Table 1). Three of these proteins demonstrated common changes in abundance at both timepoints; Fibrinogen alpha chain isoform 1 and haemoglobin subunit alpha-1/2 had increased and C9 Protein had decreased abundance across time i.e. at one day and seven days post-injury (Table 1). Supplementary proteomic Table 2 and 3 detail all the proteins identified with ≥2 peptides and any ratio between SCI and sham counterparts at 24 h and seven days post-injury, respectively.

**Table 1:** Protein changes in rat blood following contusion spinal cord injury compared to sham injury at either 1 day or 7 days following injury.

	Name	Protein ID	1 day SCI: 1 day sham	7 days SCI: 7 day sham	FDR (*p* value) (a) 1 day SCI: 1 day sham; (b) 7 day SCI: 7 day sham
Up in SCI	Fibrinogen alpha chain isoform 1	FGA	8.3	10.5	(a) 0.0007; (b) 0.0005
	Haemoglobin subunit alpha-1/2	HBA-1	8.3	3.8	(a) 0.0002; (b) 0.001
	Beta-2-glycoprotein 1	APOH	3.5		0.03
	Angiotensinogen	AGT/SERPINA8	2.6		0.02
	Immunoglobulin kappa light chain variable region	IGKV		12.9	0.03
	Prealpha-2-macroglobulin	A2M		10.8	0.03
	Fibrinogen beta chain precursor	FGB		7.9	0.02
	Myosin-11	MYH-11		6.0	0.04
	Fibrinogen gamma chain	FGG		5.75	9.48 × 10^−9^
	Serine protease inhibitor A3M	Serpina3m		3.4	0.001
	Antithrombin-III	SERPINA1		2.6	0.0002
	LOC500183 protein	LOC500183		2.5	0.03
	Interleukin-1 receptor accessory protein isoform b	IL1RAPb		2.4	0.01
	Heparin cofactor 2	SERPIND1		2.4	0.01
	Gamma-2a immunoglobulin heavy chain	Igg-2a		2.4	0.002
	Attractin	ATRN		2.3	0.04
	Immunoglobulin gamma-2b, partial	Igh-1a		2.2	0.006
Down in SCI	Antithrombin-III	SERPINC1	−3.9		2.72E-05
	Complement component 4, gene 2	C4b	−3.5		0.04
	Gelsolin	GSN	−3.4		0.0002
	Complement C5	C5	−3.3		2.07E-08
	C9 protein	C9	−2.8	−8.5	(a) 0.0001; (b) 6.87E-05
	Serum amyloid P-component	APCS	−2.5		0.002
	Plasma kallikrein	KLKB1	−2.4		0.005
	Ab1-018	Ab1-018		−20.5	0.0003
	C-reactive protein	CRP		−16.1	0.005
	Alpha-1-acid glycoprotein	ORM1		−13.7	0.01
	Vitamin D-binding protein	Gc		−9.8	1.17E-09
	C4b-binding protein alpha chain	C4BP1		−9.6	1.99E-05
	Inter-alpha-inhibitor H4 heavy chain	ITIH4		−9.4	3.88E-05
	Apolipoprotein E	APOE		−8.1	0.0002
	Alpha-1-antiproteinase	SERPINA1		−7.8	0.0004
	Complement factor H	CFH		−7.5	1.77E-11
	Complement component C6	C6		−6.3	6.26E-06
	Fibronectin 1	FN1		−5.9	1.80E-07
	Major urinary protein	Mup		−5.8	0.002
	Complement C1S subcomponent	C1s		−5.8	0.0009
	Plasma protease C1 inhibitor	SERPING1		−5.5	0.0008
	Clusterin	Clu		−4.9	0.009
	Apolipoprotein C-I	APOC1		−4.5	0.02
	Apolipoprotein B-100	APOB		−4.1	6.44 × 10^−14^ 6
	Fetub protein	FETUB		−3.5	0.001
	Apolipoprotein A-I preproprotein	Apoa1		−3.0	0.008
	Angiotensinogen	Agt		−2.7	0.027
	Afamin, isoform CRA_c	Afm		−2.6	9.02 × 10^−8^
	Complement C4	C4		−2.3	0.004
	Lumican	LUM		−2.2	0.05
	Complement component C7	C7		−2.2	6.08 × 10^−5^
	Pigment epithelium-derived factor precursor	SERPINF1		−2.1	0.002
	Vitamin K-dependent protein S	PROS1		−2.0	0.002

*FDR* false discovery rate, *SCI* spinal cord injury.

### Differential abundance of proteins in the complete versus sham injured animals

Supplementary proteomic table 5 details all the identified proteins following iTRAQ proteomic analysis of plasma from the complete SCI and sham counterpart animals. In serum collected at 14 days post injury, a comparison of complete SC injured compared to sham injured rats identified seven proteins that were differentially abundant (±2.0 FC), three of which increased in abundance after SCI (Table 2). Twelve serum proteins demonstrated a differential abundance at 112 days after a SCI compared to sham injury, with two proteins showing increased abundance and 10 showing decreased abundance (Table 2). Supplementary proteomic tables 6 and 7 detail all the proteins identified with ≥2 peptides and any ratio between SCI and sham counterparts at 14 days and 112 days post-injury, respectively.

**Table 2:** Protein changes in rat blood following complete spinal cord injury compared to sham injury at either 14 days or 112 days following injury.

	Protein	Protein ID	14 day SCI: 14 day Sham	112 day SCI: 112 day Sham	FDR (*P* value)(a) 14 day SCI: 14 day sham; (b) 112 day SCI: 112 day sham
Up after SCI	14-3-3 protein zeta/delta	YWHAZ	14.5		0.03
	Ceruloplasmin	CP		2.0	0.001
	Serine protease inhibitor A3K	SERPINA3k	2.1		0.04
	Serine protease inhibitor A3L	SERPINAA3L	2.1		0.008
	Serum amyloid P-component	APCS		1.6	0.04
Down after SCI	Actin, cytoplasmic 2	ACTG1		−3.2	0.0001
	Alpha-1-inhibitor 3	A1i3	−3	−4.6	(a) 0.0001; (b) 2.2 × 10^−6^
	Alpha-2-HS-glycoprotein	AHSG	−3.6		0.009
	Apolipoprotein A-IV	APOA4		−2.6	8.1 × 10^−6^
	Apolipoprotein B-100	APOB		−2.1	0.001
	Fibrinogen beta chain	FGB		−1.5	0.007
	Fibrinogen gamma chain	FGC		−1.6	0.006
	Fibronectin	FN1	−2.9	−4.1	(a) 1.3 × 10^−7^; (b) 6.2 × 10^−11^
	Gelsolin	GSN		−2.3	0.04
	Murinoglobulin-1	Mug1		−3	4.5 × 10^−5^
	Murinoglobulin-2	Mug2		−1.7	0.0008
	Vitamin D-binding protein	GC	−4.7		7.9 × 10^−5^

*FDR* false discovery rate, *SCI* spinal cord injury.

### Biological functions associated with the protein changes identified

In an aim to better interpret what the protein changes related to in terms of biological function, pathway analysis was performed. Biological pathways which had significant numbers of differentially abundant proteins connected with them (determined by Fisher’s exact test) could be identified.

**Fig. 1:**
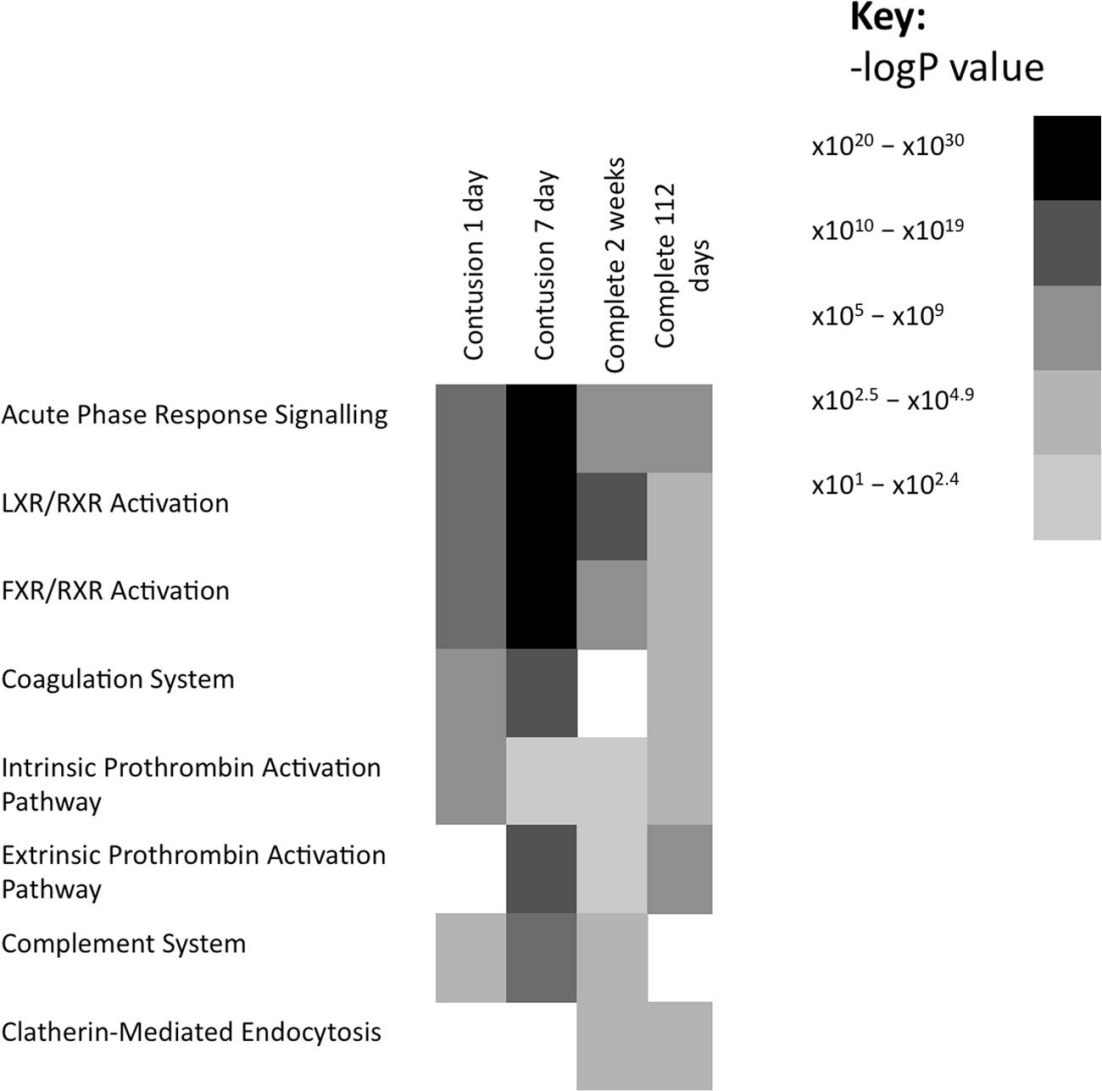
Heat map demonstrating canonical pathways that were most significantly enriched in spinal cord injured compared to sham injured rats. The significance of the association of a given canonical pathway and the differentially abundant proteins was measured using Fisher’s exact test. The heat map demonstrates the strength of the –log *P* value related to the Fisher’s exact test for each pathway in each biological comparator group.

Several functional pathways were associated with the protein changes identified in the plasma of rats with a contusion SCI compared to sham injured rats in the acute (1 day) phase of injury (Fig. 1). The most significant functional pathways were: acute phase response signalling (*p* = 1.1 ×  10^−13^; predicted inhibition); liver X receptor/retinoic X receptor (LXR/RXR) Activation (*p* = 1.8 × 10^−10^); coagulation system (*p* = 9.6 × 10^−9^) and intrinsic prothrombin activation pathway (*p* = 1.1 × 10^−6^). Several of these pathways were still effected at a more sub-acute phase of injury (7-days post-injury); namely, LXR/RXR activation (*p* = 6.0 × 10^−17^), acute phase response signalling (*p* = 1.6 ×10^−13^) and the coagulation system (*p* = 7.2 × 10^−15^). Other pathways, however, were indicated as altered only at seven days post-injury, such as the extrinsic prothrombin activation pathway (*p* = 5.9 × 10^−12^) (Fig. 1).

The same analyses were performed based on the protein changes detected within the serum of rodents with a complete SCI. Pathways that were associated with protein changes seen at two weeks post-injury included LXR/RXR activation (*p* = 3.5 × 10^−12^) and acute phase-response signalling (*p* = 3.0 × 10^−9^), the complement system (*p* = 4.1 × 10^−4^) and Clatherin-mediated endocytosis signalling (*p* = 4.27 × 10^−4^) (Fig. 1). Clatherin mediated endocytosis was also altered based upon protein changes seen at 112 days following a complete injury (*p* = 1.1 × 10^−4^), along with acute phase response signalling (*p* = 1.5 × 10^−8^), the extrinsic prothrombin activation pathway (*p* = 3.0 × 10^−5^) and the coagulation system (*p* = 1.5 × 10^−4^) (Fig. 1).

These biological pathways may provide targets for future work aimed at targeting and/or developing therapies for SCI specific responses. Furthermore, these analyses help to convey whether specific biological functions are altered over time.

## Discussion

We have presented one of the first studies to assess how the blood proteome changes after SCI in both contusive and complete transection SCI models. This investigation has presented a unique opportunity to determine the blood protein response to SCI in order to greater understand the mechanisms underlying SCI and repair.

One of the limitations of this study is that blood samples were collected from animals at different timepoints following SCI and different blood fractions were collected (plasma and serum). Therefore, this study does not aim to directly compare between the two different models but rather to demonstrate that following SCI there are protein changes within the blood, which can be detected in either blood fraction: serum or plasma. The range of timepoints in these pre-clinical models has allowed for the blood proteome to be assessed longitudinally, from the acute phase (1 day post-contusion SCI) to the chronic phase (112 day post-complete SCI) of injury. These proteomic analyses highlight that there is a differential response to SCI compared to sham injury, which can be detected in the blood in these rodent models over-time.

Pathway analyses were used to provide a better understanding of what the proteome changes might relate to in terms of biological response to injury. These analyses allow for the assessment of biological functional changes which present a more global response within the blood. Therefore commonality in functional pathway changes across time, irrespective of the blood fraction and injury severity (contusion or complete) indicates that these functional pathways are likely a specific response to SCI and not sham injury and can be taken forward for further study with much greater confidence. Moreover when the results from the two rodent models are assessed independently, these novel proteomic datasets present scientists with an understanding of the systemic response to two widely used SCI models.

In the contusion model, only 11 proteins showed differential abundance, based on our selection criteria, between the injured and the sham injured animals at 24 h post-injury. This limited number of alterations based on our fold change and *p* value cut-offs, along with the large number of common proteins identified in both the injured and sham sera, indicates that many of the protein changes that do occur as an immediate response to injury could be common to both SCI and sham injuries. Of the proteins that were most highly differentially abundant in SCI compared with sham injury at this acute timepoint, several were associated with the haematological system and haemostasis e.g. fibrinogen alpha chain isoform 1; haemoglobin subunit alpha 1/2; an observation that is strengthened by our pathway analysis, which also associated protein changes in this timeframe with the coagulation system and the intrinsic prothrombin activation pathway. These findings indicate that there is a response to SCI which can be detected within the blood immediately after injury and highlights the potential of assessing proteins which are not associated with the SCI itself, e.g. neuronal or glial related protiens, but rather that there may be a ‘whole-body’, multi-organ response that is different in response to SCI compared with sham injury. Interestingly, by seven days post-injury high numbers of differentially abundant proteins were seen, many of which displayed large FC differences in abundance, thus indicating that there may be value in measuring blood proteins through the acute phase of injury.

Studying the plasma proteome of rats following a complete injury demonstrated that up to 112 days after injury protein differences can be observed between SCI and sham injured animals. These proteins can provide an insight into the potential reparative and/or degenerative processes that continue longitudinally post-SCI. As with the contusion model, our pathway analyses have highlighted that changes within the blood occur in response to SCI, such as in the extrinsic prothrombin activation pathway and the coagulation system, and this model highlights that these may go on being implicated for a long period of time following injury. We have previously demonstrated that red blood cell (RBC) measures (RBCs, haematocrit and mean cell haemoglobin) in the blood of humans with SCI at 7 ± 4 days post-SCI correlate with initial AIS motor and sensory scores [1], confirming that systemic release of red blood cell measures may be indicative of injury severity.

Changes in acute phase response signalling were identified at both the sub-acute (14 day) and chronic (112 day) timepoints following complete injury and the acute (1 day) and sub-acute (7 day) timepoints following contusion injury, highlighting that changes in the acute phase response continue longitudinally post-SCI. Acute phase response signalling is the first systemic response to trauma [16], therefore it is unsurprising that in the case of SCI, this signalling pathway is activated. This signalling cascade is triggered by inflammatory cytokines which leads to drastically changed protein synthesis by the liver and resultant release of the acute phase proteins [16]. Therefore this signalling pathway provides an attractive target for novel therapies, potentially by targeting upstream inflammatory cytokines using biologic therapies [17]. Changes in this pathway, along with identified changes in LXR/RXR activation, add strength to our previous findings [1] also highlighting the importance of considering the effect of SCI on other organ systems aside from the nervous system, such as the liver, in contributing to clinical outcome following SCI. In future studies, it would be interesting to assess whether differences in acute-phase response signalling in the different phases of injury exist between individuals with varying injury severities and/or who either do or do not show long-term neurological improvements.

This study presents the first unbiased assessment of the blood proteome in rodent models of SCI. A large number of proteins have been identified which, with further study, may have the potential to inform on the type and severity of injury from the acute through to the chronic phase of SCI. Moreover, we have identified biological mechanisms that are associated with these protein changes, thus providing a better understanding of the blood proteome changes that occur in response to SCI.

## Supplementary information

Legend: Supplementary material includes the detailed experimental methods used in this study.

Supplementary Proteomic Tables

## Data Availability

The mass spectrometry proteomics data have been deposited to the ProteomeXchange Consortium via the PRIDE [18] partner repository with the dataset identified PXD021137.
