# Supplementary material for: Investigation of the blood proteome in response to spinal cord injury in rodent models
Source: Spinal Cord. 2021 Oct 2;60(4):320–5. doi: 10.1038/s41393-021-00692-8 (PMC8989679; doi:10.1038/s41393-021-00692-8)
Supplement: Supplementary file 1 — Legend: Supplementary material includes the detailed experimental methods used in this study. [file 41393_2021_692_MOESM1_ESM.pdf]

APPENDIX 1:

Legend: Supplementary material includes the detailed experimental methods used in this study.

DETAILED METHODS

Animals used in the study

Bloods were obtained from rats prepared for an ongoing study of the effects of SCI on the cardiovascular system, to be reported elsewhere. The analyses performed in these studies were independent of the work presented here, addressing two different biological hypotheses (one for each model). Blood samples were collected surplus to the study outcomes and were therefore used in this study to maximise the output from animal experiments in the spirit of the 3R's of animal use (replacement, refinement and reduction) [1]. Male Wistar rats were acquired from Harlan Laboratories, Loughborough, UK. Rats were 200–250 g in weight (approximately 10 – 12 weeks old), at the time of surgery and were housed in pairs under a 12-hour light/dark cycle with *ad libitum* access to food and water. All experimental procedures were approved by the Ethical Review Panel of the University of Glasgow and carried out in accordance with the Animals (Scientific Procedures) Act 1986 and adhering to our recommended good practice for SCI rodent models [2]. Bloods were obtained from animals receiving either a contusion injury performed at the C6 level or a complete spinal cord transection performed at the T9 level. Control blood samples were obtained from animals that underwent sham surgery performed at the same spinal level.

Spinal cord contusion procedure

Contusion injuries were performed using an Infinite Horizon impactor (Precision Systems Instrumentation) in the same manner as we described previously [3] . Animals were anesthetised with isoflurane and a laminectomy performed to expose the spinal cord at the C5/6 segmental level. The vertebral column was stabilized using Adson forceps and a midline impact delivered (force 175 kdyn, max velocity, 0 dwell). The wound was closed and animals recovered in warmed cabinets overnight. Sham animals were prepared by carrying out all aspects of the surgery on the animals but without impacting the cord. All animals received

analgesia (buprenorphine, 0.05 mg/kg and carprofen, 5 mg/kg, s.c., at induction of anaesthesia and the morning after surgery). Saline (3–5 ml) and enrofloxacin (5 mg/kg) were given s.c. for 3 and 7 days, respectively, after injury. Our previous work demonstrates the consistency of the injury applied using this methodological approach in terms of force and displacement [3,4], and further the consistency in functional outcome following injury (by analysing the data from animals treated with a vehicle control) [4].

Spinal cord transection procedure

Rats were anesthetised with isoflurane and a laminectomy performed to expose the spinal cord at the T9 segmental level. Spinal cord transections were performed by a method similar to that described by Lu et al. (2014) [5]. These injuries resulted in a very consistent, complete permenant hind-limb motor paralysis which consisted longitudinally. A small opening was made in the dura and the spinal cord cut transversely, at two locations, separated by approximately 1 mm, using iridectomy scissors (FST, No. 15002-08). A blunt 23G needle, connected to an aspirator, was used to remove a small amount of spinal cord tissue and any accumulating fluid. The completeness of the transection was confirmed visually by observing complete separation of the proximal and distal stumps through an operating microscope. The wound was closed and rats recovered in warmed cabinets (26˚C) for up to 3 days, until thermoregulation was restored. Sham animals were prepared by carrying out the same surgery but omitting the transection. Rats received analgesia (buprenorphine, 0.05 mg/kg and carprofen, 5 mg/kg subcutaneously at induction of anaesthesia and the morning after surgery). Saline (3–5 ml) and enrofloxacin (5 mg/kg) were given subcutaneously for 3 and 7 days after injury, respectively. Bladders were manually expressed as necessary three times daily until reflexive emptying returned (typically ten to twelve days after injury). Some of the work resulting from the preliminary experimental design is described by Williams et al. (2020) [6].

Blood Collection

Bloods were obtained from a total of 10 animals with a contusion injury; 5 were obtained at 1 day after the injury and 5 from a separate set of animals 7 days after injury. Control bloods

were obtained from 5 animals for each matched timepoint. Blood samples were also obtained from a total of 16 rats after spinal transection; 8 were obtained at 14 days after transection and 8 were obtained at 112 days after transection, as well as, control bloods from 8 animals at each timepoint. Blood was obtained by transcardial puncture. Animals were terminally anaesthetised with euthatal and when deeply anaesthetised the heart exposed by opening the chest. A 18 gauge needle was used to obtain blood from the left ventricle. Blood was processed as either serum (contusion model) or plasma (complete transection model) as these samples were required for the primary experiments. This design also allowed us to determine whether proteome changes could be identified between SC injured and sham injured counterparts in either or both blood fractions. Experimental analysis of the bloods which was completed in this study was done using excess material, so as to minimse the number of animals used. Serum and plasma was stored at -80^{0}C.

Rat blood sample preparation for isobaric Tag for Relative and Absolute Quantitation proteomics

Blood samples collected from the rodent models of SCI were assessed via two independent iTRAQ proteomic experiments. For the contusion model of SCI, 20 µl of serum was taken from individual animals and pooled to a total volume of 100 µl for the following groups: 24 hours following a 175KD weight drop (1 day SCI; n=5); 24 hours following a C6 sham injury (1 day sham; n=5); 7 days following a 175KD weight drop (7 day SCI; n=5); 7 days following a C6 sham injury (7 day sham; n=5). For the model of complete SCI, 20 µl of plasma was taken from individual animals and pooled to a total volume of 160 µl for the following groups: 14 days following a complete transection of the SC (14 day SCI; n=8); 14 days following a sham injury (14 day sham; n=8); 112 days following a complete transection of the SC (112 day SCI; n=8); 112 days following a sham injury (112 day sham; n=8).

Pooled samples were prepared for iTRAQ proteomic analysis whereby the proteins were precipitated in six volumes of ice-cold acetone overnight at -20˚C. The acetone precipitates were pelleted by centrifugation at 13,000xg for 10 mins at 4˚C and then pellets re-suspended in 200 µl triethylammonium bicarbonate buffer. The protein concentration in each of the

pooled samples was determined using a PierceTM 660 nm protein assay (Thermo Scientific, Hemel Hempstead, UK) [7]. The extracts were digested with trypsin (5 µg per 100 µg of protein) overnight at 37°C, followed by reduction, alkylation and labelling steps performed according to manufacturer's instructions (iTRAQ 8-plex assay kit, Sciex, Warrington, UK). For the contusion injury model labels were: tag 114- 1 day sham; tag 115- 1 day SCI; tag 116- 7 days sham; tag 117- 7 days injured. For the complete injury model the labels were: tag 114- 112 day sham; tag 115- 14 day sham; tag 116- 14 day SCI; tag 117- 112 day SCI. The labelled digests were then pooled and dried down in a vacuum centrifuge.

The samples were analysed at the BSRC St. Andrews University Mass Spectrometry and Proteomics Facility. iTRAQ- labelled peptides were resuspended in 0.6 mL of loading Buffer Ascx (10 mM monopotassium phosphate (KH_2PO_4), 20% acetonitrile (MeCN), pH 3.0), followed by sonication. The peptides were separated by strong cation exchange chromatography as described previously [8] into 12 fractions. Each SCX fraction was analysed by nanoLC ESI MSMS using a TripleTOF 5600 tandem mass spectrometer (ABSciex, Foster City, CA) as described previously [9]. The raw mass spectrometry data files were combined and subsequently analysed using ProteinPilot 4.5 software with the Paragon™ and ProGroup™ algorithms (ABSciex) against the rat sequences in the Swiss-Prot database. Searches were performed using the pre-set iTRAQ settings in ProteinPilot. Trypsin was selected as the cleavage enzyme and MMTS modification of cysteines with a "Thorough ID" search effort. ProteinPilot's Bias correction was used, which assumes that most proteins do not change in abundance. Finally, detected proteins were reported with a Protein Threshold [Unused ProtScore (confidence)] >0.05 and used in the quantitative analysis if they were identified with two or more peptides with >95% confidence. A False Discovery Rate (FDR) analysis was also performed using ProteinPilot software. P-values for the iTRAQ ratios were calculated by the ProteinPilot software and those with $p < 0.05$ were considered statistically significant. Proteins with iTRAQ ratios of ≥±1.2 fold change (FC) between the matched SCI and sham injured cohorts were used for network and pathway analysis to allow for identification of maximal biological pathways [10,11], whereas to assess the most pronounced differences across groups those with ≥±2.0FC were considered.

Pathway analysis of proteomic datasets

Proteins were analysed using the pathway enrichment tools in Ingenuity (Qiagen, US) [12] to identify and visualise the canonical pathways which are differentially effected between SCI and sham injured rats at each of the timepoints of the study and in the different SCI models. Functional annotations that were assigned a p-value >0.05, as assessed using a Fisher's exact test were removed from the list.

References

1. National Centre for the Replacement Refinement and Reduction of Animals in Research. Research Review. 2013. p. 1–5.

2. Lilley E, Andrews MR, Bradbury EJ, Elliott H, Hawkins P, Ichiyama RM, et al. Refining rodent models of spinal cord injury. Exp Neurol [Internet]. 2020;328(December 2019):113273. Available from: https://doi.org/10.1016/j.expneurol.2020.113273

3. Hosseinzadeh S, Lindsay SL, Gallagher AG, Wellings DA, Riehle MO, Riddell JS, et al. A novel poly-E-lysine based implant, Proliferate®, for promotion of CNS repair following spinal cord injury. Biomater Sci. 2020;

4. Lindsay SL, Toft A, Griffin J, M. M. Emraja A, Barnett SC, Riddell JS. Human olfactory mesenchymal stromal cell transplants promote remyelination and earlier improvement in gait co-ordination after spinal cord injury. Glia. 2017;65(4):639–56.

5. Lu P, Graham L, Wang Y, Wu D, Tuszynski M. Promotion of Survival and Differentiation of Neural Stem Cells with Fibrin and Growth Factor Cocktails after Severe Spinal Cord Injury. J Vis Exp. 2014;(89):1–9.

6. Williams JA, Windmill JFC, Tanner KE, Riddell JS, Coupaud S. Global and site-specific analysis of bone in a rat model of spinal cord injury-induced osteoporosis. Bone Reports [Internet]. 2020;12(November 2019):100233. Available from: https://doi.org/10.1016/j.bonr.2019.100233

7. Smith PK, Krohn RI, Hermanson GT, Mallia AK, Gartner FH, Provenzano MD, et al. Measurement of protein using bicinchoninic acid. Anal Biochem. 1985;150(1):76–85.

8. Fuller HR, Mandefro B, Shirran SL, Gross AR, Kaus A, Botting CH, et al. Spinal muscular atrophy patient iPSC-derived motor neurons have reduced expression of proteins important in neuronal development. Front Cell Neurosci. 2016;9(506).

9. Fuller HR, Slade R, Jovanov-Milošević N, Babić M, Sedmak G, Šimić G, et al. Stathmin is enriched in the developing corticospinal tract. Mol Cell Neurosci. 2015;

10. Hulme CH, Wilson EL, Peffers MJ, Roberts S, Simpson DM, Richardson JB, et al. Autologous chondrocyte implantation-derived synovial fluids display distinct responder and non-responder proteomic profiles. Arthritis Res Ther. 2017;19:150.

11. Hulme CH, Stevens A, Dunn W, Heazell AEP, Hollywood K, Begley P, et al. Identification of the functional pathways altered by placental cell exposure to high glucose: Lessons from the transcript and metabolite interactome. Sci Rep. 2018;8(1):1–28.

12. Ingenuity Qiagen. Ingenuity Knowledge Base. 2014.
